# Physicians’ Relief Fund—Final Report

**Published:** 1872-03-15

**Authors:** 


MEDICAL EXAMINER.

*A Semi-Monthly Journal of Medical Sciences.*

EDITED BY

N. S. DAVIS, M. D., AND F. H. DAVIS, M. D.

Chicago, March 15th, 1872.

### EDITORIAL.

### PHYSICIANS' RELIEF FUND—FINAL REPORT.

The committee appointed in October last to receive and distribute such contributions as might be made in money, books, or instruments, by the profession elsewhere, for the relief of those members of the regular profession here who had been placed in need by the great fire of October 9th, having completed the work assigned to them, made their final report to a public meeting of the profession held in the Criminal Court room on Monday evening, March 25th, 1872. The meeting was not large, but sufficient for the object. Dr. S. Wickersham was appointed Chairman, and Dr. A. C. Savage Secretary. The Secretary of the Medical Relief Committee, Dr. Walter Hay, gave a brief history of the work of the Committee, stating that the gross amount of money received from all sources and paid over to the Treasurer of the Committee, was $9,881.08. Of this sum, $32.50 had been used for the expenses of the Secretary, in paying for stationery, postage, and express charges. The remaining $9,848.58 had been appropriated to the relief of 91 practicing physicians, and 21 medical students, in sums varying from $30 to $350. The money had not been distributed all at once, but the Committee had met and apportioned it from time to time, as often as a sufficient sum accumulated in the treasury. In addition to the money, the Secretary had received about 70 volumes of medical books, 8 pocket cases of surgical instruments, 2 galvanic batteries, a few miscellaneous second-hand instruments, and 40 tons of coal—the latter from Dr. J. H. Rauch, of this city—all of which had been distributed except 14 tons of the coal, which remained on hand. Dr. N. S. Davis, Treasurer of the Committee, reported that he had received, at various times since the appointment of the Committee, the following sums of money:

| | | | | |
|---|---|---|---|---|
| From the physicians of | N. Y. City, | total, | $5,800 | 00 |
| " " | Brooklyn, | " | 1,338 | 00 |
| " " | Philadelphia, | " | 1,115 | 00 |
| " " | Cincinnati, | " | 457 | 00 |
| " " | St. Louis, | " | 100 | 00 |
| " " | San Francisco, | " | 253 | 08 |
| " " | Washington, | " | 200 | 00 |
| " " | Boston, | " | 157 | 00 |
| " " | Lowell, Mass., | " | 105 | 00 |
| " Rensselaer Co., N.Y., Med. Soc'y, | | " | 75 | 00 |
| " Boyle " Ky., " " | | " | 35 | 00 |
| " Physicians in various parts of the country, in small sums, | | " | 246 | 00 |
| | | Total Receipts, | $9,881 | 08 |

The Report further showed that the Treasurer had paid in accordance with the orders of the Committee, to 91 practicing physicians and 21 students of medicine, the sum of $9,835.00, and to the Secretary of the Committee for his necessary expenses in postage, express charges, and stationery, $32.50. Total paid out, $9,857.50, leaving a balance now in the hands of the Treasurer of $13.58; which the Treasurer said he would take the responsibility of giving to the first physician in good standing among the sufferers by the great fire, who should be disabled by sickness, or otherwise placed in need.

In justice to the physicians of St. Louis, it was stated that, in addition to the $100 included in the above statement of receipts, they had promptly sent $900.00 soon after the fire, and distributed it by a committee of their own. Both Secretary and Treasurer presented written vouchers for all moneys and other articles received and distributed.

On motion, Drs. Wagner, Loverin, and Savage were appointed a Committee to audit the accounts of the Secretary and Treasurer.

They did so, and reported them to be correct.

Dr. Davis alluded feelingly to the death of Drs. A. G. Morgan and Young, both of whom were burned out, and having been indisposed previous to the fire, their demise was undoubtedly hastened by the calamity.

The President spoke of the Relief Committee, saying they had done their work admirably—better than any other persons could have done it.

After tendering a vote of thanks to them, and to the physicians who contributed so generously for the relief of their Chicago brethren, the meeting adjourned.

Summer Medical Instruction.—The regular spring and summer course of instruction in the Chicago Medical College, will commence on Monday, the first day of April, and continue until the first day of July. The course will consist of three didactic lectures and examinations in the College, and from one to two hours of clinical instruction in the Hospital, each day, by members of the College Faculty. It is strictly supplementary to the winter course, extending the College instruction to *nine* months of the year, and is *free* to all the matriculated students of the College. The tickets of admission to the clinics of the Mercy Hospital are $6, and those of the Cook County Hospital $5, and both are good for one year from the date of their issue.

American Medical Association.—We call the attention of all our readers to the following circular in relation to railroad fares and hotel expenses for the coming Annual Meeting of the Association:

AMERICAN MEDICAL ASSOCIATION.
Wm. B. Atkinson, M. D.,
*Permanent Secretary*,
1400 Pine Street (S. W. cor. Broad),
Philadelphia.

The Twenty-third Annual Session will be held in Horticultural Hall, Broad Street above Spruce, on Tuesday, May 7, 1872, at 11 A. M.

HOTEL ARRANGEMENTS.

Continental, Chestnut and 9th, $4 a day; Girard, Chestnut and 9th, $3 a day; La Pierre, Broad below Chestnut, $3 a day; Colonnade, Chestnut and 15th, $3 a day; St. Cloud, Arch below 8th, $3 a day; St. Elmo, Arch above 3d, $2.50 a day; American, Chestnut below 5th, $2.50 a day; Merchants', 4th above Market, $2.50 a day; St. Lawrence, Chestnut below 12th, $2 a day; Alleghany, Market below 9th, $1.75 a day; St. Charles, 3d below Arch, lodging only 50 cents a day; Miller's, 7th and Chestnut, lodging only $1.50 a day; meals at restaurant of Horticultural Hall, and Petry's, N. W. cor. Broad and Walnut, each 50 cents.

BOARDING HOUSES.

No. 318 South Broad, $2 a day, or $10 a week; N. E. cor. Broad and Spruce, $1.50 a day, or $10 a week; 329 South Broad, $2 a day, or $10 a week; 1327 Spruce, $2 a day, or $12 a week; 225 South Broad, $2.50 a day, or $12 a week.

RAILROADS.

Union Pacific, return free, if first-class tickets are bought, and an acknowledgment taken from the agent; Cumberland Valley, excursion tickets; Orange, Alexandria and Manassas, half fare for return; Pittsburg, Cincinnati, and St. Louis, excursion tickets; Pittsburg, Fort Wayne, and Chicago, excursion tickets; Cleveland and Pittsburg, excursion tickets; Central Railroad of Georgia, return free; Richmond and Petersburg, return free; Wilmington and Weldon, excursion tickets one fare; Wilmington, Columbia, and Augusta, excursion tickets one fare; Kansas Pacific, one and one-fifth fare for excursion; Atlanta and New Orleans Short Line (A. and W. Pt. Western, Mobile, and M. N. O., M. and Texas Railroads), return free; Western and Atlantic, excursion tickets one fare; Western Alabama, excursion tickets one fare; Evansville and Crawfordsville, excursion tickets; Lehigh Valley, excursion tickets one fare; Louisville and Nashville, excursion tickets; Memphis and Louisville, excursion tickets; North Pennsylvania, excursion tickets two-thirds fare; Pennsylvania Central, excursion tickets; Philadelphia and Erie, excursion tickets; Philadelphia, Wilmington, and Baltimore, excursion tickets; Philadelphia and Reading, excursion tickets at two-thirds; Baltimore and Ohio, excursion tickets; Lake Shore and Michigan Southern, excursion tickets if forty are taken.

All who desire to avail themselves of the above rates, must send to the Secretary their full names, and the names of *all* the railroads over which they must travel in coming to the session, with stamp for postage.

Wm. B. Atkinson.

SPECIAL.

Camden and Amboy, excursion tickets at $4 from New York to Philadelphia and return, if fifty tickets are taken. For this ticket, send money to Dr. A. E. M. Purdy, 123 East Thirtieth Street, New York.

From Montgomery, Ala., to Philadelphia, and return (by Tennessee), $39.80. Apply through Dr. R. F. Michel, Montgomery, Ala.

From Washington to Philadelphia and return, $6, if fifty tickets are taken.

Central Pacific, half local rates.

THE WESTERN LANCET.—This is the title of a new monthly medical journal published at San Francisco, the first number of which is on our table. It makes a good appearance, and we cheerfully place it on our list of exchanges.

THE LENS; a Quarterly Journal of Microscopy and the Allied Natural Sciences. The first number of this valuable journal is before us. It is published under the auspices of the State Microscopical Society of Illinois, and edited by S. A. Briggs. It is to be issued quarterly, contains 64 pages of reading matter, and is well illustrated. It will be a periodical of special value to all who are interested in microscopical investigations.

